# The Purpose of Nursery Schools

**Published:** 1920-05-29

**Authors:** 


### THE PURPOSE OF NURSERY SCHOOLS.

Estimates have been before the Buildings Sub-Committee of the London Education Committee for the necessary alterations required by a modest experimental scheme of nursery schools; three such schools are to be attached to existing elementary schools in Bethnal Green, Mile End, and Paddington. There exists a good deal of vagueness as to what the nursery school is actually intended to be, and for that reason it is useful to give the definition contained in the memorandum of the Board of Education. It says: "A nursery school or class is an institution providing for the care and training of young children aged from two to five years, whose attendance at such a day school is necessary or desirable for their healthy physical and mental development. It has, therefore, a two-fold function: first, the close personal care and medical supervision of the individual child, involving provision for its comfort, rest, and suitable nourishment; and, secondly, definite training—bodily, mental, and social—involving the cultivation of good habits in the widest sense, under the guidance and oversight of a skilled and intelligent teacher and the orderly association of children of various ages in common games and occupations."

The nursery school is to be much more than a place for looking after children. The medical supervision will form a very important side of the work done, and one reason for the provision of nursery schools is to reduce the large number of preventable defects now observed in entrants to the public elementary schools and the associated educational handicap and resulting incapacity. For several years past the degree and character of defects prevalent among children on their first admission to the elementary schools have revealed a widespread measure of low physical condition in children under five, not a little of which might have been prevented if it had been properly dealt with between two and five years of age.

### Association with Day Nurseries.

There is a feeling in some quarters (says *The Times*) that the nursery school should be as near as possible to, if not actually run in connection with, existing day nurseries and *crèches*. Otherwise the mother who works will find it a very difficult problem to leave her young children at different centres which may be a considerable distance from each other and open at different times, before she goes to her own work. This point should have due consideration with the education authorities. The memorandum provides that children in attendance at a day nursery may defer admission to a nursery school until they reach the age of three years, and suggests that, while the accommodation in nursery schools is likely to be limited for some time, the interests of the young children should, as a rule, take precedence of children between the ages of five and six.

When the nursery schools are fully established there will be a chain of medical supervision and record from these schools right through the elementary schools and to the end of the secondary schools. The school medical inspection hitherto carried out has not yet provided the ameliorative results that might have been expected from it, and one of the explanations is, most probably, that inspection does not begin early enough. The nursery schools' inspection should remedy that defect to some extent. But even so, the first two years of life are very precious and important in relation to the whole future life of the child, and medical supervision here is nothing like complete.

